# Revisiting the Metabolic Capabilities of *Bifidobacterium longum* susbp. *longum* and *Bifidobacterium longum* subsp. *infantis* from a Glycoside Hydrolase Perspective

**DOI:** 10.3390/microorganisms8050723

**Published:** 2020-05-13

**Authors:** Guillermo Blanco, Lorena Ruiz, Hector Tamés, Patricia Ruas-Madiedo, Florentino Fdez-Riverola, Borja Sánchez, Anália Lourenço, Abelardo Margolles

**Affiliations:** 1 Escuela Superior de Ingeniería Informática, Edificio Politécnico, Campus Universitario As Lagoas s/n, University of Vigo, 32004 Ourense, Spain; guillermo@guillermoblanco.es (G.B.); riverola@uvigo.es (F.F.-R.); 2 Department of Microbiology and Biochemistry of Dairy Products, Instituto de Productos Lácteos de Asturias (IPLA), Consejo Superior de Investigaciones Científicas (CSIC), Paseo Río Linares S/N, Villaviciosa, 33300 Asturias, Spain; hector.tames@ipla.csic.es (H.T.); ruas-madiedo@ipla.csic.es (P.R.-M.); borja.sanchez@csic.es (B.S.); amargolles@ipla.csic.es (A.M.); 3 Instituto de Investigación Sanitaria del Principado de Asturias (ISPA), Oviedo, 33011 Asturias, Spain; 4 CINBIO-Centro de Investigaciones Biomédicas, University of Vigo, Campus Universitario Lagoas-Marcosende, 36310 Vigo, Spain; 5 SING Research Group, Galicia Sur Health Research Institute (IIS Galicia Sur), SERGAS-UVIGO, Hospital Álvaro Cunqueiro, 36312 Vigo, Spain; 6 CEB-Centre of Biological Engineering, University of Minho, Campus de Campus de Gualtar, 4710-057 Braga, Portugal

**Keywords:** *Bifidobacterium*, *longum*, *infantis*, carbohydrates, glycoside hydrolases, computational screening

## Abstract

Bifidobacteria are among the most abundant microorganisms inhabiting the intestine of humans and many animals. Within the genus *Bifidobacterium*, several beneficial effects have been attributed to strains belonging to the subspecies *Bifidobacterium longum* subsp. *longum* and *Bifidobacterium longum* subsp. *infantis*, which are often found in infants and adults. The increasing numbers of sequenced genomes belonging to these two subspecies, and the availability of novel computational tools focused on predicting glycolytic abilities, with the aim of understanding the capabilities of degrading specific carbohydrates, allowed us to depict the potential glycoside hydrolases (GH) of these bacteria, with a focus on those GH profiles that differ in the two subspecies. We performed an in silico examination of 188 sequenced *B. longum* genomes and depicted the commonly present and strain-specific GHs and GH families among representatives of this species. Additionally, GH profiling, genome-based and 16S rRNA-based clustering analyses showed that the subspecies assignment of some strains does not properly match with their genetic background. Furthermore, the analysis of the potential GH component allowed the distinction of clear GH patterns. Some of the GH activities, and their link with the two subspecies under study, are further discussed. Overall, our in silico analysis poses some questions about the suitability of considering the GH activities of *B. longum* subsp. *longum* and *B. longum* subsp. *infantis* to gain insight into the characterization and classification of these two subspecies with probiotic interest.

## 1. Introduction

Bifidobacteria are a group of ubiquitous microorganisms, whose main habitat includes different animals, including mammals, some insects and birds, although they have also been isolated from food and environmental samples, among other sources [1]. Several species can be found in humans and are considered among the first colonizers of the gut, constituting one of the most abundant genera in the fecal microbiota of healthy individuals [2]. Among them, *Bifidobacterium longum*, *Bifidobacterium breve*, *Bifidobacterium bifidum*, *Bifidobacterium animalis*, and *Bifidobacterium adolescentis* have received a QPS (Qualified Presumption of Safety) designation according to EFSA (European Food Safety Authority) [3]. For this reason, these QPS bifidobacterial species have been widely studied as potential probiotics and some strains are included in a large variety of food or food supplements due to the proven health benefits attributed to specific strains [4]. It is widely accepted that *B. longum* is currently subdivided into three subspecies: *B. longum* subsp. *longum, B. longum* subsp. *infantis*, and *B. longum* subsp. *suis* [5], although a fourth subspecies (*suillum*) has recently been proposed [6]. While the subspecies *suis* and *suillum* are not common in humans, they are mainly found in piglet feces, subspecies *longum* and *infantis* are very common in children, especially in breast-fed infants, the subspecies *longum* also being one of the most frequently detected bifidobacteria in the microbiota of healthy adult individuals [7,8].

Representatives of the subspecies *longum* and *infantis* were the first bifidobacteria to be fully sequenced [9,10]. *B. longum* is currently the species for which the highest number of fully sequenced genomes is available in public databases, the large majority of them belonging to the subspecies *longum* and, to a lesser extent, *infantis*. The extensive genetic information currently available for these two subspecies has allowed studies of comparative functional genomics that indicate some strategies used for these microorganisms to colonize the gastrointestinal tract. Among them, it is worth highlighting its ability to use a large amount of glycan rich sources. It is noteworthy that a large percentage of gene families in the *Bifidobacterium* pangenome are associated with carbohydrate metabolism, which allows these bacteria to metabolize host and diet derived carbohydrates that are not digested by the host and give them an adaptive advantage in the intestine. In general, genetic and functional studies seem to indicate that the subspecies *infantis* is better adapted to the intestinal environment of nursing children because it is capable of metabolizing human milk oligosaccharides (HMOs) [10,11,12]. Nonetheless, the carbohydrate metabolic capabilities in the subspecies *longum* seem to be more oriented to metabolize complex vegetable carbohydrates, and do not have the same ability as the subspecies *infantis* to degrade HMOs [13,14]. Within the enzymatic arsenal responsible for carbohydrate metabolism, glycoside hydrolases (GHs) constitute one of the main players and are responsible for the cleavage of complex carbohydrates, i.e., cleaving glycosidic bonds in poly- and oligosaccharides, and releasing shorter metabolizable products. However, the genetic information available in the databases does not always establish clearly different genetic patterns between the two subspecies. 

In this work, we took advantage of novel computational tools to depict the GH capabilities of *B. longum* subsp. *longum* and *B. longum* subsp. *infantis*, with a specific focus on those activities that differentiate the two subspecies, with the aim of contributing to understanding metabolic traits that partially explain their differences in carbohydrate utilization.

## 2. Materials and Methods:

### 2.1. Phylogenetic Trees Based on 16S rRNA, ldh, recA, and tuf Genes 

*B. longum* 16S rRNA gene sequences with a length >1500 bp and a sequence quality >90% were downloaded from the SILVA database in FASTA format [15]. Only the strains with assigned *longum* or *infantis* subspecies were used in the analysis. Additionally, complete and partial sequences of the genes *ldh*, *recA,* and *tuf* were analyzed. For multiple sequence comparison, Clustal Omega (version 1.2.4) was used for alignment [16]. Basically, a single plain text file containing all sequences was uploaded using HHalign package, via pairwise comparisons. Progressive alignment was carried out using a hidden Markov model (HMM) and iterative clustering steps. Finally, multiple reference phylogenetic trees were built up (via neighbor joining) and using data from pairwise comparison, trees were refined until finally one reference tree met criteria. Phylogenetic tree information was pasted into a plain text file again, and visualized in iTOL v5 (interactive Tree of Life) [17]. 

### 2.2. Whole Genome Phylogenetic Tree

From all *B. longum* completed and closed genomes available in the National Center of Biotechnology Information (NCBI) database, only the genomes with assigned *longum* or *infantis* subspecies were considered for phylogenetic tree building. Complete genome sequences were retrieved from the NCBI database without annotations. In order to enable a consistent comparative analysis, first open reading frame predictions and gene annotations were performed using PROKKA v1.12 [18]. Then, comparative genomics and phylogenetic tree construction were created using a ROARY pipeline [19]. Basically, GFF3 sequence outputs from Prokka were preclustered all against all using BLASTP and MCL (Markov Cluster), and after the preclustering step, further iterative comparisons between sequences with identity lower than 100% were performed using BLAST and clustering using CDhit algorithm. After grouping similar sequences in clustering steps, paralogs were split using conserved gene neighborhood (CGN) to finally distinguish between core, soft-core, shell, and cloud genes based on prevalence. All this information was used to create the phylogenetic tree using FastTree. Finally, iTOL was used for visualization of phylogenetic tree [17].

### 2.3. Genome Sequences of B. longum

To obtain protein files of *B. longum* subsp. *infantis* and *B. longum* subsp. *longum*, the Gleukos tool was used [20]. Gleukos automatized the process of extracting and decompressing protein files of *Bifidobacterium* strains. With this tool we were able to retrieve genomic information from the NCBI genomic database and collect 188 genomes of different strains of *B. longum*, 171 of them had translated protein files (147 genomes of subspecies *longum*; 24 genomes of subspecies *infantis*). Supplementary File 1 includes a summary of the genomic information of each strain.

### 2.4. In Silico Analysis of B. longum GH Capabilities

The first step in this analysis entailed the compilation of the GHs of *B. longum* subsp. *longum* and *B. longum* subsp. *infantis.* The completed list of GHs is displayed in Suplementary File 2. Different data sources were used to gather together gene and protein information, namely the Carbohydrate-Active Enzymes database (CAZy) [21], the Kyoto Encyclopedia of Genes and Genomes (KEGG) [22], and the Genome database of NCBI [23]. To automatize this process, the Gleukos tool was used [20].

The glycolytic profiles of the reference strains were retrieved from the available genomes in KEGG. In particular, reference strains include three genomes of *B. longum* subsp. *infantis* (two of them belong to the same strain that has been sequenced in two different sequencing projects), i.e., *B. longum* subsp. *infantis* ATCC 15697 JGI (identified as “bln”), *B. longum* subsp. *infantis* ATCC 15697 Tokyo (identified as “blon”), and *B. longum* subsp. *infantis* 157F (identified as “blf”). Additionally, six strains of *B. longum* subsp. *longum* were analyzed as reference strains, i.e., *B. longum* subsp. *longum* JDM301 (identified as “bll”), *B. longum* subsp. *longum* BBMN68 (identified as “blb”), *B. longum* subsp. *longum JCM* 1217 (identified as “blm”), *B. longum* subsp. *longum* KACC 91563 (identified as “blk”), *B. longum* subsp. *longum* F8 (identified as “blg”), and *B. longum* subsp. *longum* GT15 (identified as “blz”). For each of these strains, all annotated proteins were screened, keeping only GHs for further analyses. In total, this screening resulted in 417 GH protein matches.

The previously described 417 proteins were matched against all the genomes of *B. longum* strains with associated protein files. The tool BLASTp (version 2.7.1) [24] enabled the large-scale screening of glycolytic activities. Specifically, the BLAST database, containing the previously described 417 proteins, was built by executing the command “makeblastdb” with the parameter “dbtype prot”. The command “blastp”, with the parameters “outfmt 6” and “max_target_seqs 1”, enabled the matching of all the genomes of *B. longum* strains against this database (i.e., 171 strains with associated protein files). These results were represented in a heatmap created with the Gleukos using InCHlib library [25], in which the Y axis represents the GH proteins and the X axis represents the collection of *B. longum* strains. Furthermore, each axis included a dendrogram with the corresponding hierarchical clustering of its data. These hierarchical clusters were built considering the Euclidean distance as intra-cluster proximity metric and average linkage as inter-cluster distance. Heatmap coloring represented the scoring of protein homologies, i.e., red indicates <50% identity and green >50% identity (with at least 50% identity across at least 50% of either protein sequence) [26].

## 3. Results and Discussion

**Figure 1:**
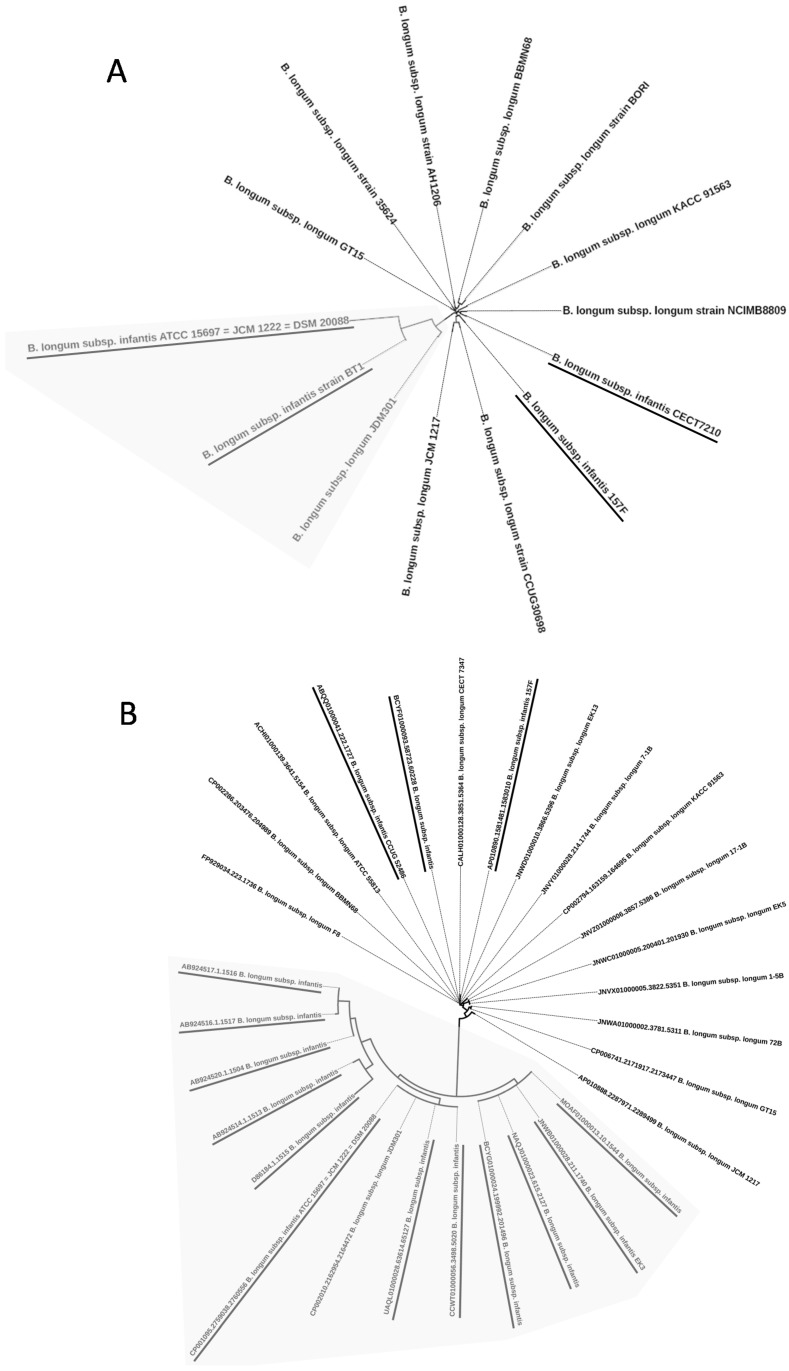
Genome-based (**A**) and 16S rRNA gene-based (**B**) clustering analysis. *Bifidobacterium longum* subsp. *infantis* strains are underlined. The clusters rich in *B. longum* subsp. *infantis* strains are represented with grey background.

In order to discern the global genetic and evolutionary variability among annotated *B. longum* subsp. *longum* and *B. longum* subsp. *infantis*, a clustering analysis was performed based on different genetic traits, including 16S rRNA gene and whole genome sequences. Phylogenetic analysis allowed us to construct two trees with strains of *B. longum* subsp. *longum* and *B. longum* subsp. *infantis*, whose complete genomes or 16S rRNA genes are available in public databases (Figure 1). First, a tree was built based on the comparison of complete genomes that had been annotated in the databases with the corresponding subspecies assignment (Figure 1A). Subsequently, a tree was constructed taking into account the sequences of the 16S rRNA gene, using only the sequences available in the SILVA database of those strains for which a subspecies has been assigned (Figure 1B). Furthermore, a clustering analysis considering full or partial sequences of *recA*, *tuf*, and *ldh* genes, previously used for subspecies identification in *B. longum* [27,28,29,30], was also carried out with the strains included in Figure 1A (Supplementary File 3). Neither of the trees have included strains for which the subspecies is not specified. That is, all the strains deposited as “*B. longum*”, without specifying the subspecies, were not taken into account in our analysis.

When comparing the complete genomes, two clearly defined clusters are observed, the most populated including 11 strains mainly belonging to the subspecies *longum*. The second one, consisting of three strains, includes two strains of *infantis* and one of *longum*. This result seems to indicate that there is no good correlation between the comparative genomic analysis and the corresponding subspecies assignment, since in the group dominated by strains belonging to the subspecies *longum*, strains deposited as *B. longum* subsp. *infantis* are also present (see CECT 7210 and 157F). Meanwhile, one *B. longum* subsp *longum* strain (JDM301) is also found in the group with the majority of *infantis* strains. The *B. longum* subsp. *infantis* strains CECT 7210 and 157F are also included in branches dominated by *B. longum* subsp. *longum* strains when complete and partial sequences of the genes *recA* and *tuf* are used for the clustering analysis (Supplementary File 3). Therefore, this highlights that there could be certain discrepancies when assigning subspecies to strains that are genetically closer to the other subspecies. This lack of harmony between the genetic profile and the subspecies assignment in some strains is also evident when *Bifidobacterium* strains were clustered according to their rRNA 16S ribosomal gene sequence (Figure 1B).

Previous comparative genomics studies have already reported that some *B. longum* subsp. *infantis* strains might have been taxonomically misassigned [13,31]. O´Callaghan and coworkers carried out a phylogenetic analysis of 33 complete and incomplete *B. longum* strains and showed that *B. longum* subsp. *infantis* 157F is a member of the subspecies *longum* [13]. In our work, we also showed that the strain *B. longum* subsp. *infantis* CECT 7210 clusters together with other strains of the subspecies *longum*, and it is most likely a member of the *longum* subspecies. This strain, isolated from infant feces, provided in vivo protection against rotavirus infection in a mouse model [32]. Thus, our data suggest that care must be taken when comparing *B. longum* genomes deposited in public databases, since our results and the results of others show that subspecies assignment might have not been accurate for some strains. However, to avoid confusion, the probable misclassifications were not corrected in the subsequent comparative analysis of the GH families.

**Figure 2:**
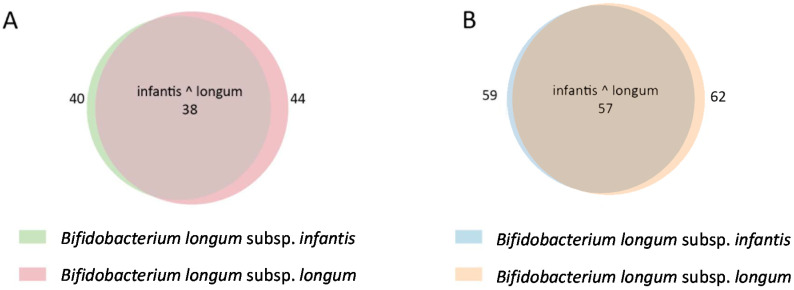
Venn diagram representing the distribution of glycoside hydrolases (GHs) in the *Bifidobacterium longum* reference strains (**A**) and the distribution of GH families (**B**). The numbers in the overlapping areas correspond to shared GHs (**A**) or GH families (**B**).

In this context, we hypothesized that genetic traits relevant for bifidobacterial adaptation to the gastrointestinal tract ecosystem of different population groups, might add relevant information for *B. longum* subspecies classification. Indeed, traditionally, it has been documented that the metabolism of complex sugars is a differential trait of these two subspecies. In this regard, the metabolism of *infantis* seems to be oriented to the degradation of the oligosaccharides found in human milk, whereas the subspecies *longum* lacks this ability and focuses its carbohydrate metabolic potential in the utilization of complex vegetable carbohydrates obtained from diet [10,11,13,14,33,34]. Glycoside hydrolases (GHs), enzymes responsible for cleaving glycosidic bonds in sugar polymers, releasing shorter oligo- and polymers or sugar moieties, play a key role in many of the steps of the carbohydrate metabolic processes in bacteria [20]. A comparative analysis of the presence of GHs in the reference strains of these two subspecies (the nine reference *B. longum* strains previously defined and for which KEGG and functional annotations were publicly available), enabled us to detect 46 different GHs, 38 of them were found in both subspecies, and the results are shown in Figure 2 and Supplementary File 2. Furthermore, these 46 different GHs were distributed across 64 GH families, 57 of them being present in both subspecies. Figure 2 shows Venn diagrams displaying the distribution of different GHs and their family allocation by subspecies.

According to the Glycoside Hydrolase Family Classification (CAZY), and considering the reference strains, GHs from the families GH8, GH70, GH72, GH79, and GH94 are only present in the subspecies *longum*, and GH34 and GH83 have only been found in the subspecies *infantis*. Supplementary File 2 details the matching of these families and the different subspecies *longum* and *infantis*. Regarding specific GHs, the presence of EC 2.4.1.230 (kojibiose phosphorilase) and EC 3.2.1.18 (sialidase) was unique for *infantis*, and EC 2.4.1.4 (amylosucrase), EC 3.2.1.31 (beta-glucuronidase), EC 3.2.1.41 (pullulanase), EC 3.2.1.45 (glucocerebridase), EC 3.2.1.99 (arabinanase), and EC 3.2.1.156 (reducing end xylose-releasing exo-oligoxylanase) were only found in *longum*. These enzymes represent a GH spectrum that can allow the selection of specific carbohydrates preferentially metabolized by each subspecies. Indeed, some GH substrates containing the specific bonds hydrolyzed by these enzymes, have already been reported as preferential carbon sources for *longum* or *infantis*. This is the case of sialidases, since sialic acid decorates the structure of HMOs, whose specific metabolism has been associated with *infantis* [11]. Additionally, vegetable oligo and polysaccharides containing arabinose, xylose, or both, have been previously used to promote the growth of *B. longum* subsp. *longum*, as well as other *Bifidobacterium* species [35,36]. However, our analysis also showed kojibiose as a potential novel substrate specific for *infantis*. Kojibiose is a disaccharide product of glucose caramelization, composed of two glucose molecules linked through an alpha 1–2 bond, which is present in honey, beer, and other foods, normally in low amounts. Interestingly, the prebiotic and bifidogenic activity of kojibiose and some kojibiose-derivatives have been previously studied [37], but their potential to specifically favor the growth of *infantis* and not that of *longum*, to the best of our knowledge, has not been explored.

**Figure 3:**
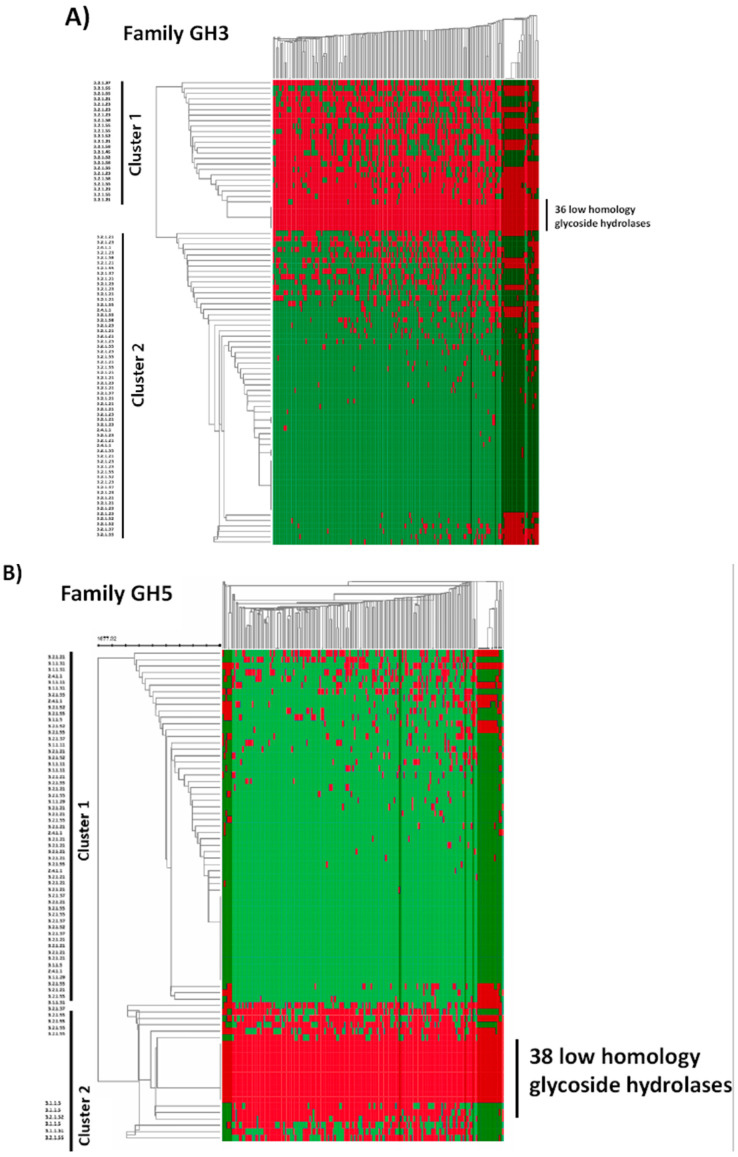

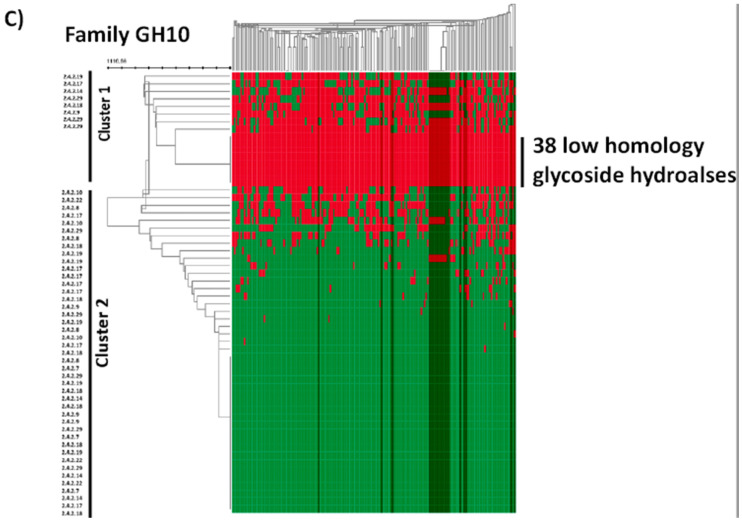
Heatmaps of the GH3 (**A**), GH5 (**B**), and GH10 families (**C**). The X axes represent 171 *B. longum* strains, and in the Y axes the different GH proteins are included. The red colors indicate homologies (protein level) below 50%, and the green color indicates that the homology is higher than 50%. The *B. longum* subsp. *infantis* strains are represented in darker columns.

**Figure 4:**
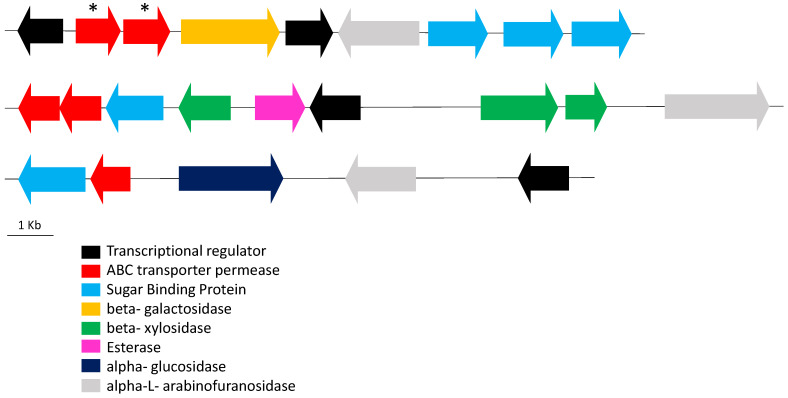
Representation of the different gene clusters containing alpha-l-arabinofuranosidases from family E.C. 3.2.1.55 in the genome of *B. longum* subsp. *longum* F8. *, genes not annotated in the genome of the F8 strain, but annotated as “ABC transporter permease” in the genomes of the strains JDM 301, BBMN68, JCM 1217, KACC 91563, and GT15.

In an attempt to perform a more rational analysis of the GH capabilities of *B. longum,* we used Gleukos, a new bioinformatics tool that may be of help in a wide range of biotechnological applications [20]. This new public Web portal integrates the CAZY and KEGG information and contributes with insights by extending the search scope of potential glycolytic sources in a custom set of publicly available genomes, without the need to conduct extensive genome annotations and comparative whole genomic analysis. Additionally, Gleukos enables the identification of the GH arsenal of intestinal bacteria at different taxonomic levels. Expanding the array of bacterial glycoside-acting enzymes capable of metabolizing specific bifidogenic substrates was our aim in this analysis. To do this, the Interactive Cluster Heatmap library (InCHlib) was applied to display and cluster heatmaps [25]. As an example that illustrates the outcome of Gleukos, Figure 3 shows the heatmap for the 171 *B. longum* selected strains and the GHs belonging to the GH3 (Figure 3A), GH5 (Figure 3B), and GH10 (Figure 3C) families, three of the families containing GHs involved in the degradation of prebiotic products and widely found in *B. longum* (Supplementary File 2) and contain many enzymes. A global heatmap, considering all the GH families and the 171 *B. longum* strains, is depicted in Supplementary File 4. Additionally, an interactive heatmap showing the presence/absence of GH homologs in the 171 genomes is available in the URL http://193.147.87.218:8888/gleukos/task?id=3279. Remarkably, some of the GH enzymes associated with the subspecies *infantis* include sialidases, fucosidases, and *N*-acetyl-galactosaminidases, all of them involved in the degradation of complex HMOs [12,38,39]. In addition, EC.3.2.1.55 was the GH enzyme more frequently associated with *longum*. EC 3.2.1.55 are alpha-l-arabinofuranosidases that catalyze the hydrolysis of terminal non-reducing α-l-arabinofuranoside residues in α-l-arabinosides, frequently found as a component of hemicelluloses and pectins, and that cannot be absorbed by intestinal cells. A search within the six genomes of the subspecies *longum* strains included in the KEGG database (strains JDM 301, BBMN68, JCM 1217, KACC 91563, F8, and GT15), showed that the genes encoding these enzymes are present in the genomes organized in clusters of carbohydrate utilization. The most typical cluster structure is composed of nine genes, including genes coding for transcriptional regulators, ABC transporters, sugar binding proteins, a beta-galactosidase, and an alpha-l-arabinofuranosidase. This cluster structure is present in the six genomes analyzed, although the genome of the strain F8 also contains other alpha-l-arabinofuranosidase enzymes, organized in different clusters including other sugar-actin enzymes nearby, such as beta-xylosidases and alpha-glucosidases (Figure 4). Indeed, arabinose and xylose can often be together in arabynoxylan structures [40,41], suggesting that those bifidobacteria possessing arabinose and xylose acting enzymes would have an additional advantage to metabolize this substrate.

Several papers have reported the involvement of *Bifidobacterium* alpha-l-arabinofuranosidases in the hydrolysis of arabinans, arabinoxylans, and arabinogalactans [40,41,42,43], and the prebiotic and/or bifidogenic activity of these substrates [44,45]. Since cereals are very rich in arabinan-containing sugar polymers, the fact that alpha-l-arabinosidases are over-represented in the subspecies *longum*, but are absent or scarcely present in *infantis*, could indicate the adaptation of the subspecies *longum* to fiber-rich vegetable substrates in an adult-like diet. In this regard, it is worth noting that several authors have already demonstrated that *B. longum* subsp. *longum* representative strains are capable to degrade either arabinan or arabinoxylans [34,41,46]. Remarkably, among the bifidobacterial species with QPS status, l-arabinosidases and l-xylosidases appear to be restricted to *B. longum* subsp *longum*, suggesting that it represents a specific trait related to this bifidobactarial group that could be exploited in future biotechnological applications aimed at developing novel subspecies specific bifidogenic substrates and symbiotic formulations.

## 4. Conclusions

The work presented in this manuscript highlights that the publicly available genomes of some strains of *B. longum* subsp. *longum* and *B. longum* subsp. *infantis* clearly depicted GH patterns associated with the two subspecies. Some of the GH activities discussed in this work, such as the preference of *B. longum* subsp. *infantis* for HMOs, were already reported in literature, but other observations open new possibilities to explore the GH activities of these two subspecies, in order to favor the representativeness of one or the other subspecies in the human gut microbiota, depending on the substrate composition of the surrounding environment, as well as the design of “a la carte” oligo and/or polysaccharides specifically targeted to fulfil the metabolic capabilities of these subspecies. In addition, our findings also showed the potential of the Gleukos tool to explore GH maps in *Bifidobacterium*, a process that could be extrapolated to other bacterial groups. 

## Supplementary Materials

The following are available online at https://www.mdpi.com/2076-2607/8/5/723/s1, Supplementary File 1: Information of the *Bifidobacterium* genomes retrieved from the NCBI database for the present study; Supplementary File 2: GH and GH families considered in the current work. Green boxes indicate the presence of GH and GH families. Supplementary File 3: Clustering analysis of *B. longum* subsp. *infantis* and *B. longum* subsp. *longum* strains based on *ldh* (A,D), *recA* (B,E), and *tuf* (C,F) complete and partial sequences. Supplementary File 4: Heatmap of the different GH found in the 171 genomes of *B. longum* subsp. *infantis* and *B. longum* subsp. *longum.* The X axes represent 171 *B. longum,* and in the Y axes the different GH proteins are included. The red colors indicate homologies (protein level) below 50%, and the green color indicates that the homology is higher than 50%. The *B. longum* subsp. *infantis* strains are represented in darker columns.
