# Supplementary material for: Revisiting the Metabolic Capabilities of Bifidobacterium longum susbp. longum and Bifidobacterium longum subsp. infantis from a Glycoside Hydrolase Perspective
Source: Microorganisms. 2020 May 13;8(5):723. doi: 10.3390/microorganisms8050723 (PMC7285499; doi:10.3390/microorganisms8050723)
Supplement: Supplementary file 1 [file microorganisms-08-00723-s001.zip › Supplementary File 3 def.pptx]

## Slide 1
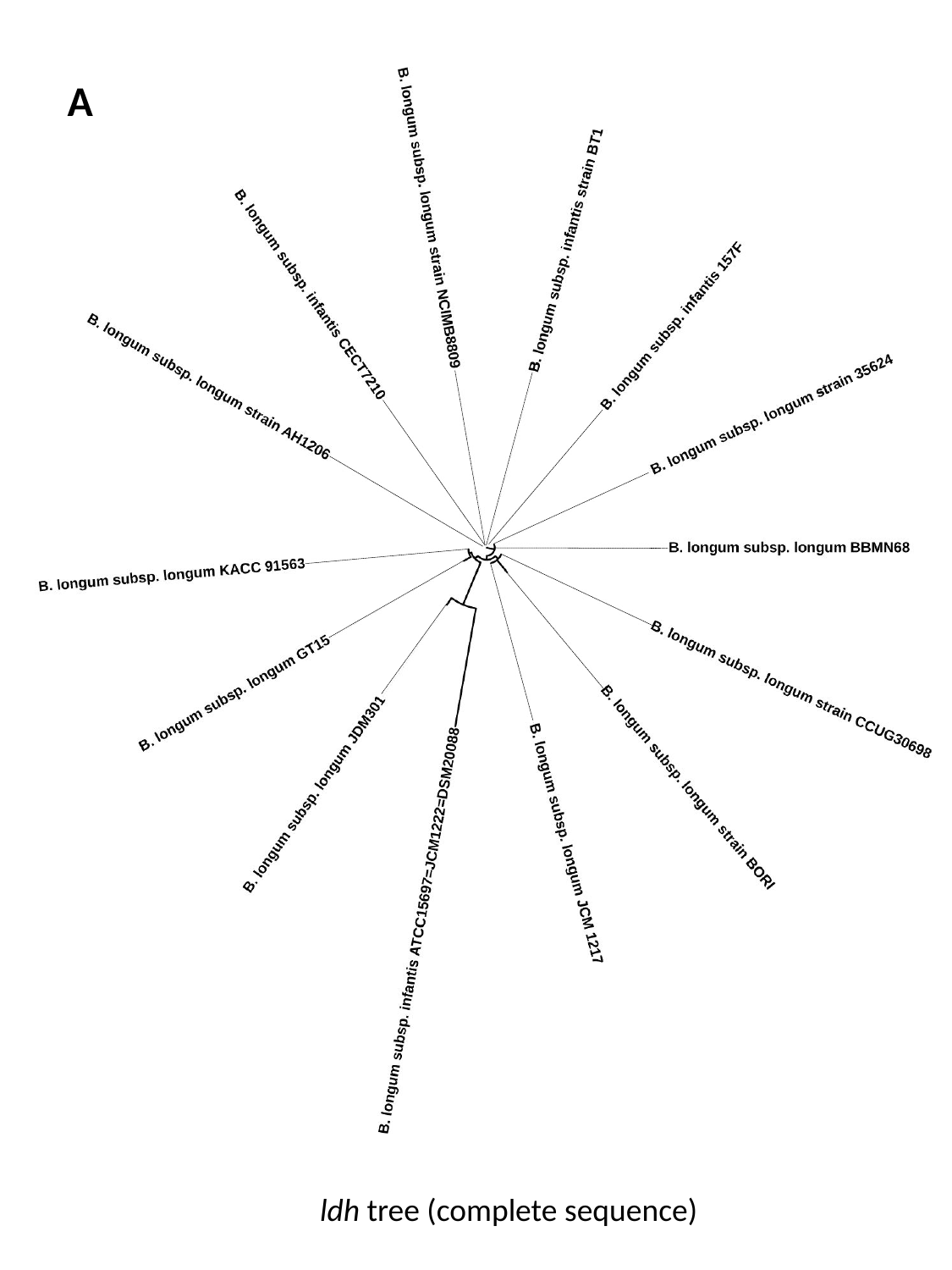

A
ldh tree (complete sequence)

## Slide 2
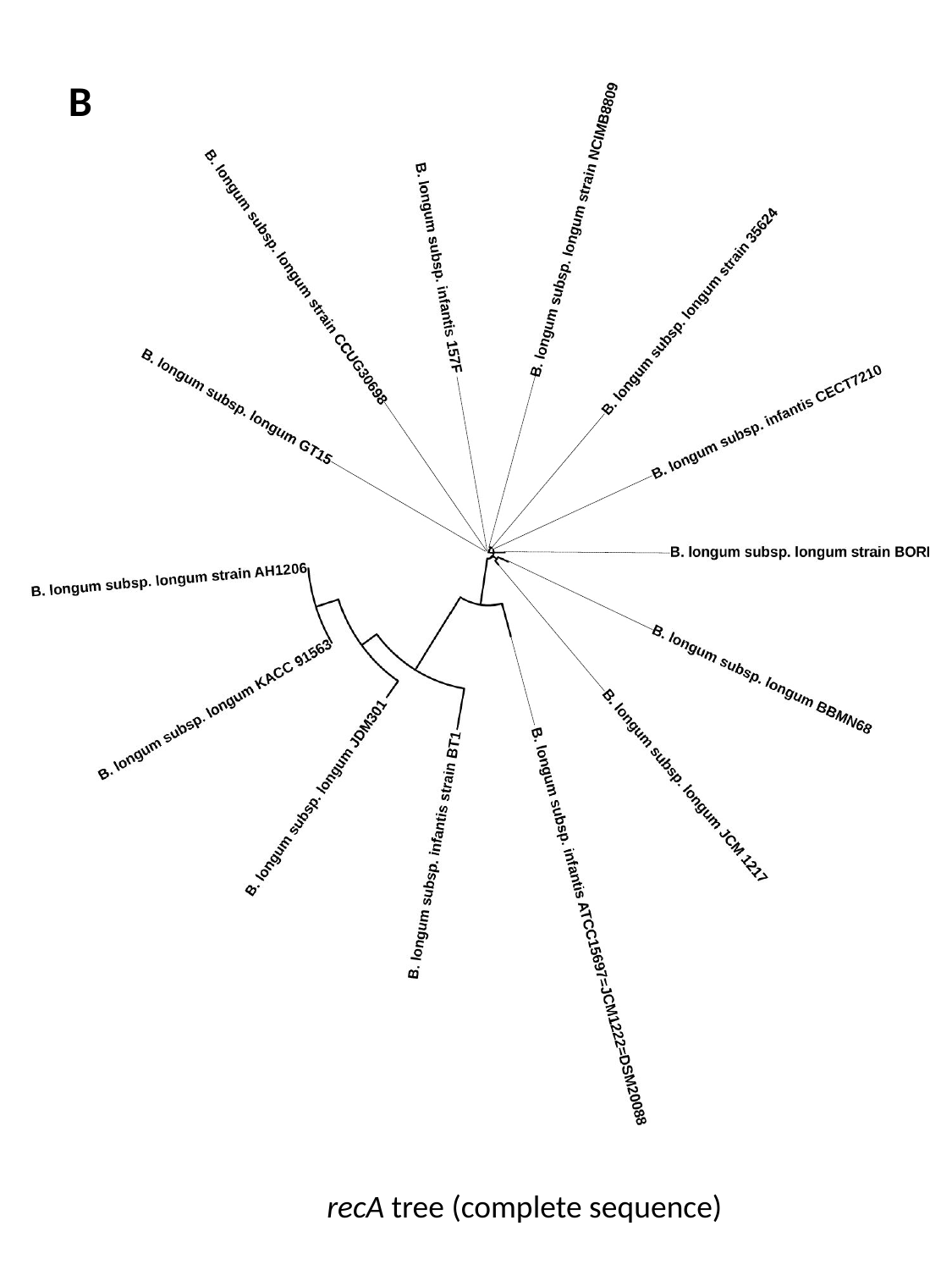

B
recA tree (complete sequence)

## Slide 3
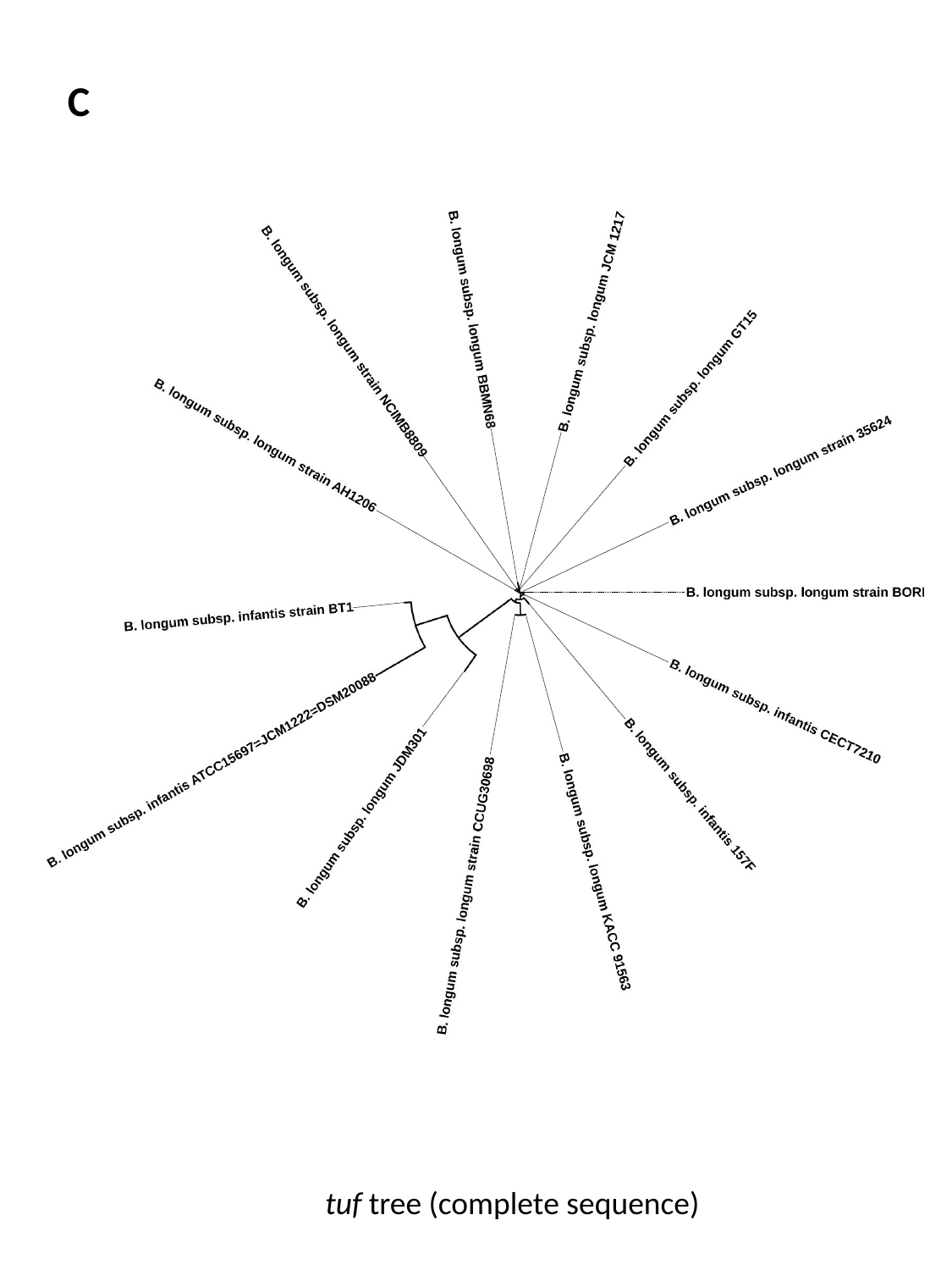

C
tuf tree (complete sequence)

## Slide 4
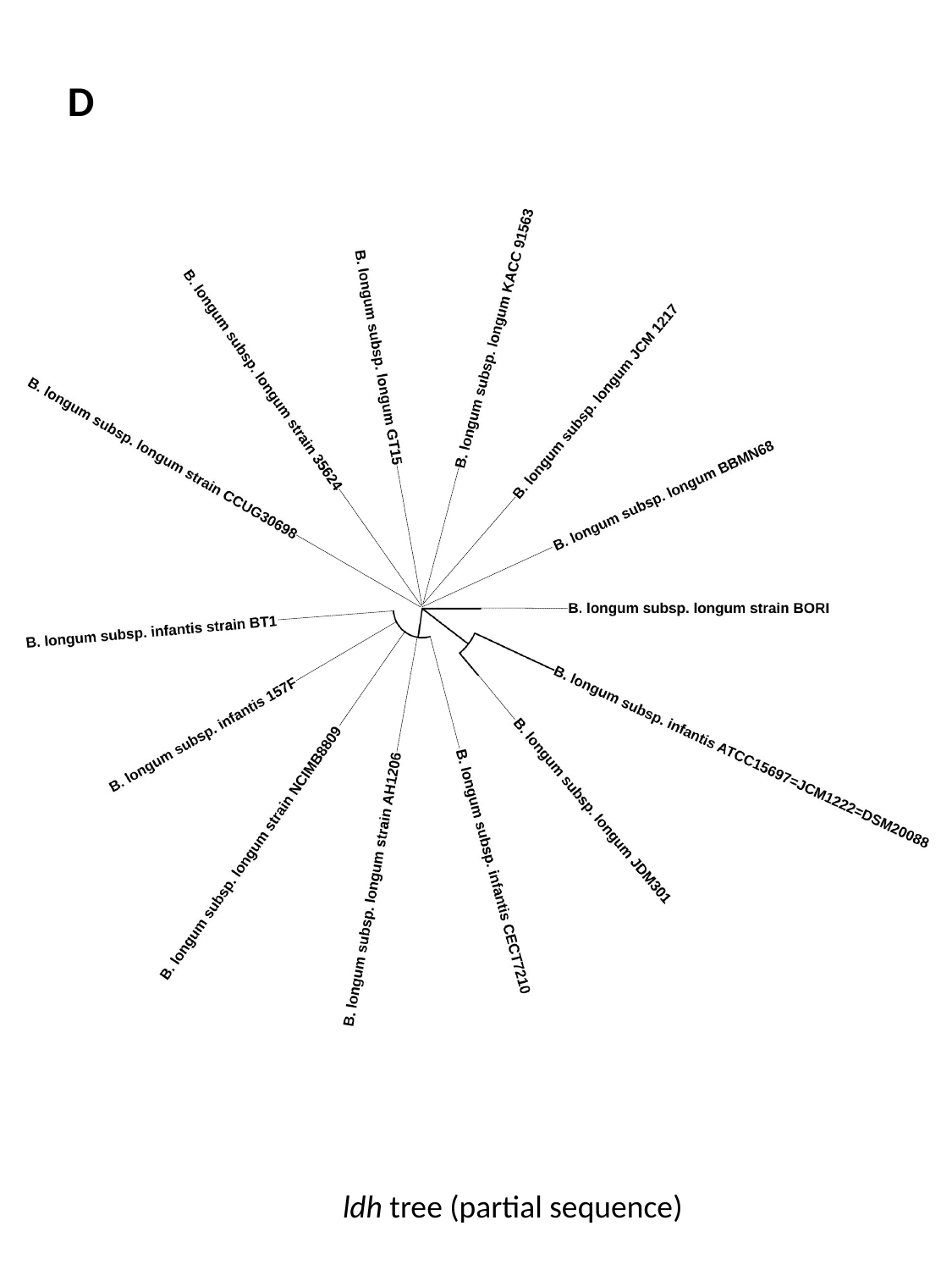

D
ldh tree (partial sequence)

## Slide 5
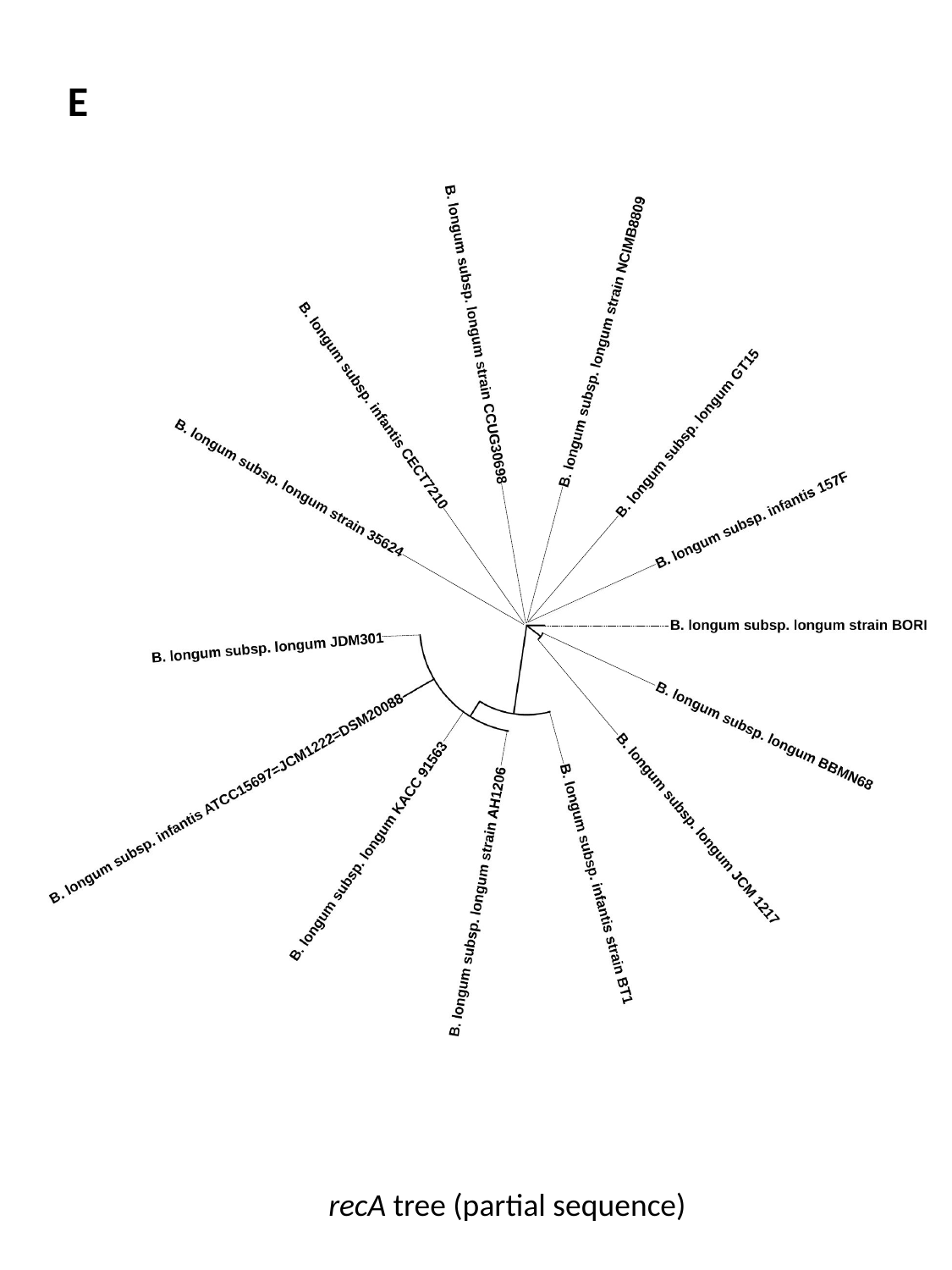

E
recA tree (partial sequence)

## Slide 6
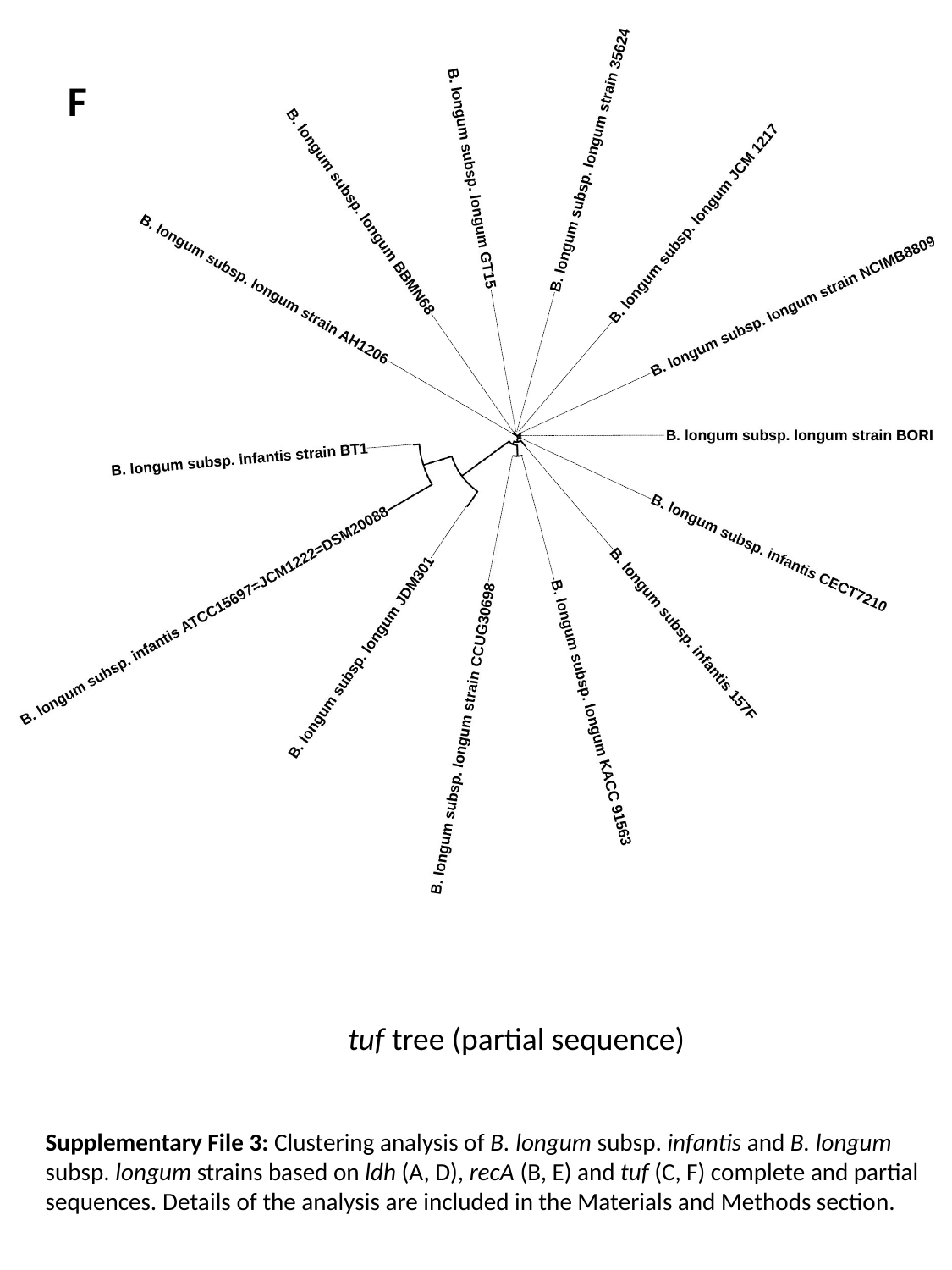

F
tuf tree (partial sequence)
Supplementary File 3: Clustering analysis of B. longum subsp. infantis and B. longum subsp. longum strains based on ldh (A, D), recA (B, E) and tuf (C, F) complete and partial sequences. Details of the analysis are included in the Materials and Methods section.
